# Bioinformatics studies of Influenza A hemagglutinin sequence data indicate recombination-like events leading to segment exchanges

**DOI:** 10.1186/s13104-016-2017-3

**Published:** 2016-04-15

**Authors:** Antara De, Tapati Sarkar, Ashesh Nandy

**Affiliations:** Centre for Interdisciplinary Research and Education, 404B Jodhpur Park, Kolkata, 700068 India; Physics Department, Jadavpur University, Jadavpur, Kolkata, 700032 India

**Keywords:** Recombination, Hemagglutinin sequences, RNA characterization, HA segment exchange

## Abstract

**Background:**

The influenza genome is highly variable due primarily to two mechanisms: antigenic drift and antigenic shift. A third mechanism for genetic change, known as copy choice or template switching, can arise during replication when, if two viral strains infect a cell, a part of a gene from the second viral strain can be copied into the growing progeny of a gene of the first viral strain as replacement leading to a new variety of the virus. This template switching between the same genes of the two strains is known as homologous recombination. While genetic drift and shift are well-understood, the presence or absence of intra-segment homologous recombination in influenza genomes is controversial.

**Context and purpose of study:**

We are interested to study the possibility of subunit-wise homologous recombination. The idea is that where well-defined subunits are separated by consensus sequences, it might be possible for template switching to take place at such junctions. The influenza hemagglutinin gene has basically two subunits, HA1 and HA2, with HA1 being mostly surface exposed and containing the active site for binding to cells, while HA2 secures the hemagglutinin to the viral coat. We undertook a thorough search of the major human infecting influenza hemagglutinin gene sequences, viz., the H1N1, H5N1, H3N2 and H7N9 subtypes, over the period 2010–2014 in Asia to determine if certain sequences could be identified that had HA1 from a previous strain and HA2 from another.

**Results:**

Our search yielded several instances where sequence identities between segments of various strains could be interpreted as indicating possibilities of segment exchange. In some cases, on closer examination they turn out to differ by a few mutations in each segment, due perhaps to the short time span of our database.

**Conclusions and potential implications:**

The study reported here, and in combination with our earlier observations on the neuraminidase, shows that subunit-wise recombination-like events in the influenza genes may be occurring more often than have been accounted for and merits further detailed studies.

**Electronic supplementary material:**

The online version of this article (doi:10.1186/s13104-016-2017-3) contains supplementary material, which is available to authorized users.

## Background

Influenza is a seasonal infectious viral disease that causes death to thousands every year and occasionally many times more in case of severe pandemics. The influenza virus belongs to the family *Orthomyxoviridae* and the genetic material is a negative sense ssRNA segmented into eight genes that code for eleven structural and non-structural proteins: hemagglutinin (HA), neuraminidase (NA), matrix proteins M1 and M2, nucleoprotein (NP), non-structural proteins NS1, NS2 and the polymerases PA, PB1, PB1-F2 and PB2 [7, 12]; within the PA gene is embedded the code for a second protein discovered recently, PA-X, accessible by ribosomal frameshifting, constituting a twelfth protein of the influenza A protein family [21, 38]. The hemagglutinin is responsible for viral entry into a cell and the neuraminidase is responsible for viral elution. The HA and NA, located on the surface of the virion, have been classified into several subtypes (H1–H18 for the hemagglutinin and N1–N11 for the neuraminidase at present [41]) depending on their antigenicity, and the influenza virus itself is named according to the cell-surface hemagglutinin and neuraminidase protein subtypes: thus the H1N1, H7N9, etc. With 18 hemagglutinin subtypes and 11 neuraminidase subtypes there could be 198 subtypes of influenza, but in nature only certain subtypes are found to be widely prevalent due to HA–NA interdependence [34].

The influenza genome is highly variable due primarily to two mechanisms: antigenic drift and antigenic shift. Antigenic drift occurs due to errors by the transcription machinery of the viral genome as the viral RNA polymerase lacks proof reading activity. This leads to accumulation of point mutations, sometimes in the antigenic domain that may give rise to new subtypes. As a result, humoral antibody produced because of previous exposure can no longer mount effective immune response and mutant strains escape immune elimination; in the right circumstances this may cause local epidemic cycles.

The second mechanism that causes viral variability and known as genetic shift is due to gene reassortment. This is specific to segmented genes and happens when different subtypes of viruses enter a single cell. The replicated segments of the various subtypes can reassemble with exchange of genes from the different subtypes and sometimes lead to totally new strains which may not have existed before and therefore can cause widespread epidemics and pandemics before the immune response can catch up with the new viral strain. It has been documented that the H7N9 China flu of 2013 was due to reassorted genes from several viral strains: HA gene from avian H7N3, NA gene from avian H7N9 and other genes derived from avian H9N2 [13, 26, 27]. The genes underwent reassortment in one unknown intermediate host and finally affected humans with very high mortality ratio; however, due to different mechanism of infection and strict containment practices this new strain has not reached epidemic or pandemic proportions.

There can, however, be a third mechanism for genetic change which comes about by a copy choice, or template switching method during replication. If two viral strains infect a cell, during replication a part of a gene from the second viral strain can be copied into the growing progeny of a gene of the first viral strain as replacement leading to a new variety of the virus. This template switching can take place between different genes, known as non-homologous recombination [35], or, more commonly, between the same genes of the two strains, i.e. homologous recombination. Homologous recombination is well documented in the case of eukaryote and bacterial genomes [29], but is controversial in cases of negative stranded RNA viruses [18]. Such viruses are believed to be rapidly packed with ribonucleoprotein after being transcribed and it then becomes difficult, but perhaps not impossible, for RNA polymerase to ‘jump’ from one strand to another to search for similar sequences [42]. In mammalian genes and chromosomes, recombination can take place through one or more cross-overs during replication [29]. Because viral polymerase only binds to few nucleotides and therefore could jump from one template to another [11], during viral replication there can be segment stretches within a genetic sequence that are copied across by template switching; the start and end of such sequences are marked by certain sequence motifs known as breakpoints.

The ability of influenza virus to mutate so rapidly and often enough to be highly pathogenic and virulent, makes it imperative to monitor these changes and understand the various mechanisms that lead to such changes in the viral genome. While genetic drift and shift are well-understood, the presence or absence of homologous recombination in influenza genomes is controversial and merit further studies [6, 17, 22–24, 44]. In a recent paper, Weilong Hao studied recombination events among 256 genes of 32 sequences of influenza A virus in silico [20]. From computer based studies he identified three recombinants derived from recombination of PB2 genes of two parents. By maximum likelihood phylogenetic analysis the author showed that the parents belonged to two clades present at two ends of the tree while the three daughter subtypes are present in-between. The phylogenetic tree showed high bootstrap values. However, Hao’s contention has been strongly contested, primarily by Boni et al. [5].

In this paper we have considered a slightly different scenario: instead of template switching back and forth between short intra-segments as described by Boni et al. [5], we considered the possibility of subunit-wise homologous recombination. The idea is that where well-defined subunit segment sequences are separated by consensus sequences, it might be possible for template switching to take place at such junctions. We note that recombination has been seen between subtypes of feline immunodeficiency virus, a DNA virus, [2] where recombinants of subtype A/B, A/C and C/D were detected in cats which suffered double infection, although this is rare in natural conditions; parental subtypes of intersubtype recombinations were also detected. Recombination has been reported among segments in certain isolates of hepatitis B virus, also a DNA virus [8]. Evidences for such recombinations in RNA viruses do not appear to be established so far. Aziz and Tempfer [1] have referred to possibilities of segment exchange recombination in RNA virus-resistant transgenic plants, but the results were not conclusive. Worobey and Holmes [43] mention that segmental exchange recombination in phi6 RNA virus is impossible or very rare. They go on to state that template switching by viral replicase may be inhibited by physical constraints such as the ribonucleoprotein packaging in the case of negative-strand RNA viruses, also corroborated by White et al. [42] as mentioned earlier. However, one other physical constraint mentioned by them, viz., the extent of sequence dissimilarity between potentially recombining genomes, is obviated in our case by our imposed condition that potential matches ensure identical sequences between parent and daughter segments. Han et al. [19] have reported evidence of possible homologous recombination in negative stranded RNA viruses such as Newcastle disease virus, Zaire ebola virus, measles virus and canine distemper virus, but dispute existence of homologous recombination in different types of influenza virus.

In our studies of mutational changes in influenza neuraminidase sequences [16], we did notice some sequence identities in segments or subunits of different strains and concluded that there exists a possibility of such subunit, or segment (terms used interchangeably in this article) exchanges, but the lead was not explored further. In the present paper we have chosen influenza hemagglutinin for our study, the choice dictated in part by the fact that several recombination studies have been done on this gene [6, 17, 23, 44]. Hemagglutinin protein on the virion surface exists as a trimer; the monomer has basically two subunits, HA1 and HA2, with HA1 being mostly surface exposed and containing the active site for binding to cells, while HA2 secures the hemagglutinin to the viral coat. The combination of HA1 and HA2 provide for efficient working of the influenza gene. In this context, exchanges of one or the other subunit/segment RNA sequences from the varieties available in cases where multiple influenza infections occur in a single individual cell might give the new varieties that survive higher stability and efficiency and lead to greater evolutionary advantage. It is possible that there is historical precedence for this phenomenon: Gibbs et al. [17] have reported a detailed study suggesting that in fact the 1918 H1N1 Spanish Flu pandemic was a result of recombination in the H1 of an avian H1N1 flu where part of the HA1 globular domain had recombined with a swine lineage flu HA1 and the stalk region, HA2, had parts derived from a human lineage hemagglutinin HA2 to produce a new strain with novel antigenic sites to which the human immunological apparatus had no defense to offer.

To determine whether total segment exchange as we have hypothesized could have taken place, we undertook a thorough search of the major human infecting influenza HA gene sequences, viz., from the H1N1, H5N1, H3N2 and H7N9 subtypes, over the period 2010–2014 in Asia. Extending the coverage to wider spans of time and geography could conceivably show more events matching our criteria, but for the present we limit ourselves to the limited database mentioned above. We considered here a simple formula to determine recombination-like events: only when a daughter segment, in this case meaning either HA1 or HA2, is found identical to the same segment in some other, previous hemagglutinin strain, then named parent 1, and the other segment is similarly found identical to some other, different strain, viz., parent 2, then we will identify such an event as a possible recombination-like event. Our task is therefore to scan all HA1 sequences and all HA2 sequences separately and based on the identical sequences found, identify the two-parents–one-daughter combinations. In this sense our definition closely parallels the “precise similarly-essential recombination” classification of Nagy and Simon [30].

This is indeed different from the established pattern of inference about recombination. Classical recombination technique would consider small stretches of sequence within the HA1 or HA2 segments and consider polymerase jumps between breakpoints that mark the start- and end- points of those short segments. Phylogenetic trees could be drawn that would show clustering of sequences that constitute one parent sequence or the other arising mainly from the fact that the sequences could be quite different overall while being identical in small parts, the distances being computed from the totality of differences between the sequences. In our case where we search for identical sequences for each complete segment, the phylogenetic trees would show a complete overlap of segment sequences and not a clustering of close sequence similarities. What we are choosing to do is a simple exercise: Determine those sequences that are identical, and therefore contain no provision for any other possibilities. Phylogenetic trees here, therefore, become redundant.

Our search yielded several instances where sequence identities between segments of various strains could be interpreted as indicating possibilities of segment exchange. In some cases, on closer examination, the parents turn out to differ by a few mutations in each segment, due perhaps to the short time span of our database. Our study here, and in combination with our earlier observations on the neuraminidase, shows that subunit-wise recombination-like events in the influenza genes may be occurring more often than we have accounted for and merits further detailed studies.

## Methods

### Data

We considered all complete sequences of the hemagglutinin gene of human infecting flu subtypes H1N1, H5N1, H3N2 and H7N9 that were reported in the period 2010–2014 from the Asian continent and documented in GenBank database (http://www.ncbi.nlm.nih.gov/genomes/FLU/Database/nph-select.cgi?go=database). The H7N9, a more recent human infecting subtype, were mostly reported from China; other subtypes were observed in various countries of Asia with H1N1 having the largest spread. The total sample size was 1274. Table 1 gives a subtype-wise breakup of the total number of sequences we analyzed.

**Table 1:** Total number of sequences analyzed and number of sequence-based recombinants

Subtype	Total no. of sequences analyzed	No. of recombinants and % of total
H3N2	408	30 (7.35 %)
H1N1	427	16 (3.74 %)
H5N1	350	20 (5.71 %)
H7N9	89	7 (7.87 %)
Total	1274	73 (5.73 %)

### Sequence identity determination

**Fig. 1:**
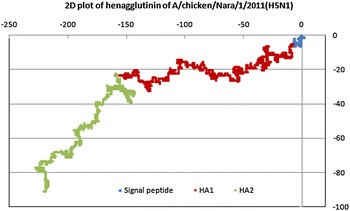
2D graphical representation of hemagglutinin of A/chicken/Nara/1/2011(H5N1) showing base distributions of the signal peptide region (*blue*), HA1 (*red*) and HA2 (*green*)

Since we were searching for segment exchange recombination which requires the daughter sequence to have HA1 from one parent and HA2 from the other, it is critical to determine exact matches between the different subunits of parents and daughter. For the 1274 sequences with average 1700 bases in each hemagglutinin sequence, that requires consideration of over 2 million bases for matching. We used the new technique of graphical representation and numerical characterization [31, 37] to compute the descriptors of each segment and compare for exact matches, the idea being that exact matches imply identical sequences. In this method a 2D graphical plot is generated for each of HA1 and HA2 subunit nucleotide sequences for each strain on a Cartesian axes system by moving one step in the negative x-direction for an adenine, one step in the positive y-direction for a cytosine, one step in the positive x-direction for a guanine, and one step in the negative y-direction for a thymine. Starting from the origin and continuing in this manner sequentially for an entire segment’s sequence creates a 2D plot of the segment that is reflective of the base distribution along the sequence. Figure 1 shows a graphical representation of the hemagglutinin component of the influenza strain A/chicken/Nara/1/2011(H5N1), GenBank ID AB684259, worked out as per the description given above. The two segments HA1 and HA2 are marked in separate colours for easy identification. Noting the axes as A, C, G, T starting from negative x-axis and going clockwise, such a plot gives a quick visual rendering of the distribution of bases in the sequence; in particular, in this instance, the distinctions between the HA1 and HA2 are quite discernible.

To numerically characterize a DNA/RNA sequence or a segment of one, we define a weighted centre of mass of the plot (*μ*_*x*_*, μ*_*y*_) and a graph radius *g*_*R*_ as follows [37]:

$${\mu _x} = \sum\limits_{i = 1}^N {{x_i}}{\big/} {N} ,\quad {\mu _y} = \sum\limits_{i = 1}^N {{y_i}}{\big/} {N}$$

$$g_{R} = \sqrt {\mu_{x}^{2} + \mu_{y}^{2} }$$

where *x*_*i*_*, y*_*i*_ represent coordinates of the *i*^*th*^ base and *N* is the total number of nucleotides in the sequence under consideration. The graph radius *g*_*R*_ is an index of the base distribution in the nucleotide sequence and is found to be sensitive to any changes in the base distribution such that sequences having same value of *g*_*R*_ imply sequence identity [33]. We have used the *g*_*R*_ index in a number of applications: We determined the spread of the H5N1 avian flu strains across the world and, by analyzing the neuraminidase gene sequences, determined through the *g*_*R*_ values locations of identical strains, and also identified where identical segments of the neuraminidase were found [16]. Close monitoring of the *g*_*R*_ values of the neuraminidase strains enabled us to identify particular regions of the neuraminidase sequences that were very highly conserved which led to determination of surface situated highly conserved stretches of the protein sequence that could be used for targeting peptide vaccines [15], and extended, through a corresponding protein graph radius, to rational design of peptide vaccines against rotavirus [14]. The concept of graph radius has been used in a more general sense in determining a hemagglutinin-neuraminidase interdependence index [34], for sequence comparisons by Jayalaksmi et al. [25], by Yao et al. [48] in analysis of similarity/dissimilarity of DNA sequences in a 3D representation, by Tang et al. [40] for computing in a 4-dimensional space the Euclidean distance between the representations of two DNA sequence, by Qi et al. [36] as one of three distance measures in comparing DNA sequences, by Das et al. [9] to assess DNA similarities in 2D and 3D systems, and by several other authors. We can thus take the graph radius as an acceptable characterization index of DNA/RNA sequences.

The basic idea of characterization of a selected length of DNA/RNA sequences to measure sequence similarities and dissimilarities is a prime motive in all graphical representation and numerical characterization methods, a very large number of which have been proposed in the last couple of decades (see review [32]). The large majority of them use matrix methods to uniquely characterize the sequences, but these are difficult to evaluate for large sequences and usually end up in approximations. The 2D graphical representation method we have described in some detail above, on the other hand, is intuitively simple and numbers are easy to compute with closure even for very large sequences. Hence this is our preferred method for computation, and, in our search for identical segments over the 1274 sequences with 2548 segments under consideration, indices like *g*_*R*_ constitute a very useful tool. One could conceivably compare the sequences available in the normal character designated form, i.e. as a, c, g, t, but the task is rendered much easier when a group of such characters is represented by a number which will change whenever there is a change in any character in the group. The *g*_*R*_ merely serves this purpose.

**Fig. 2:**
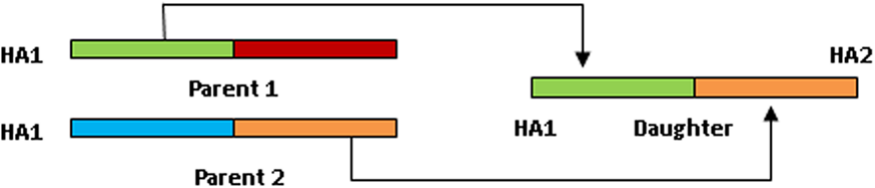
Schematic diagram of segment exchange between two hemagglutinin sequences. The HA1 (*light green*) from parent 1 and HA2 (*orange*) from parent 2 combine to form a new daughter sequence

The algorithm used was to compute the *g*_*R*_ values for HA1 and HA2 separately for all the 1274 sequences in our database and determine sequence identities pairwise for daughter sequences which had HA1 identical with one parent and HA2 identical with another. Figure 2 provides a schematic diagram of the parents-daughter relationships.

### HA1, HA2 cleavage site

Central to our hypothesis is availability of a consensus sequence where the polymerase can do a template switch during sequence replication. The HA1–HA2 cleavage site has a consensus sequence stretch of 5–6 nucleotides which remains almost conserved in the hemagglutinin RNA. Table 2 lists a sampling of 27 sequences for a stretch of 10 nucleotides around the cleavage site; the bases marked in italics span six nucleotides, three on either side of the cleavage point, and are seen to be well conserved

**Table 2:** Representative samples of 27 hemagglutinin nucleotide sequences of all subtypes considered in this work at HA1–HA2 junction point

Virus subtype	GenBank locus ID	HA1-HA2 junction	
H1N1	JF275925	ct*aga*	*ggc*ct
H1N1	CY187255	ct*aga*	*ggc*ct
H1N1	JQ065328	ct*aga*	*ggc*ct
H1N1	AB762406	ct*aga*	*ggc*ct
H1N1	AB551871	ct*aga*	*ggc*ct
H1N1	AB704815	ct*aga*	*ggc*ct
H1N1	CY056294	ct*aga*	*ggc*ct
H5N1	AB569348	aa*aga*	*gga*ct
H5N1	AB849460	aa*aga*	*gga*ct
H5N1	AB972715	aa*aga*	*gga*ct
H5N1	AB675739	aa*aga*	*gga*ct
H5N1	KF369222	ag*aga*	*gga*tt
H3N2	KJ955515	ct*aga*	*ggc*at
H3N2	KM276899	at*aga*	*ggc*at
H3N2	CY091837	ct*aga*	*ggc*at
H3N2	CY124183	ct*aga*	*ggc*at
H3N2	CY116636	ct*aga*	*ggc*at
H3N2	CY124187	ct*aga*	*ggc*at
H7N9	KC609780	ga*aga*	*ggc*ct
H7N9	CY147028	ga*aga*	*ggc*ct
H7N9	KC896763	ga*aga*	*ggc*ct
H7N9	CY147084	ga*aga*	*ggc*ct
H7N9	CY147132	ga*aga*	*ggc*ct
H7N9	KJ415822	ga*aga*	*ggc*ct
H7N9	KJ946417	ga*aga*	*ggc*ct
H7N9	KM374042	ga*aga*	*ggc*ct

Ten nucleotides are displayed, five each from HA1 and HA2, with three on either side of the junction displayed in italics. These can be seen to be very well conserved

## Results

**Fig. 3:**
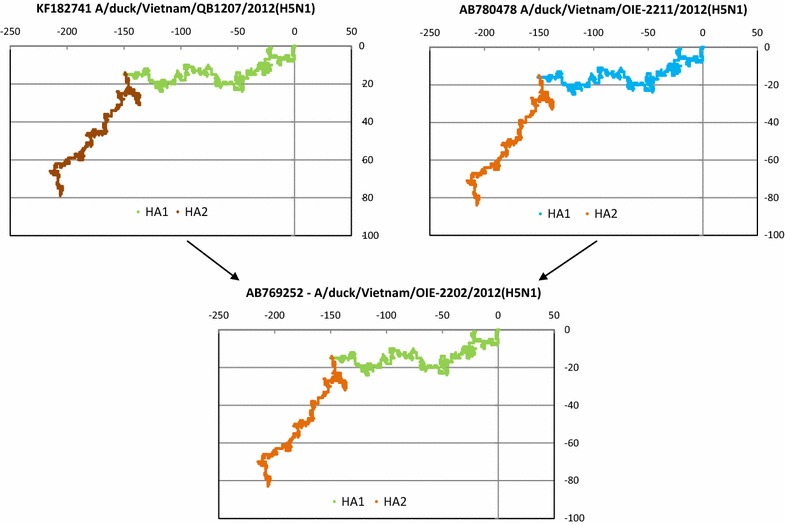
2D graphical representations of two actual parental H5N1 strains of hemagglutinin giving rise to a daughter strain following item 12 of H5N1 group in Additional file 1. *Colour schemes* are as in the schema in Fig. 2

With our algorithm described above, we found in our hemagglutinin sequence database with 1274 entries built up as mentioned in the previous section that there were 73 instances, i.e. 5.73 % of all sequences (Table 1), where a progeny hemagglutinin strain could be identified as having inherited one identical segment each from two different HA strains, which could therefore be labeled as its parents (Additional file 1). That means, out of the 1274 sequences in our analysis, 146 sequences (11.46 % of the total) could be paired to yield 73 daughter sequences each of which had a HA1 from one parent and a HA2 from the other of the matched pair. In Additional file 1 the parents-sibling are arranged in each instance as a triplet where the donor of HA1 is listed first, the donor of HA2 is listed second and the donee who gets the HA1 from parent 1 and HA2 from parent 2 is listed third. E.g., the first entry in the table for virus subtype H1N1 shows that HA1 of A/swine/Hong Kong/NS4846/2011(H1N1) and HA2 of A/swine/Hong Kong/4902/2011(H1N1) are found in identical copies in A/swine/Hong Kong/NS4848/2011(H1N1) as can be seen from the *g*_*R*_ values given in the table; the collection date is 15th December 2011 in all three cases, thereby including the possibility that they could have undergone a recombination-like event resulting in the daughter strain. A 2D graphical representation of the entry in item 12 of H5N1 sequences in Additional file 1 shows visually the segment exchange that appears to have occurred between the two parental strains and the resultant daughter strain (Fig. 3).

The second entry in the table shows an apparent incongruity: the collection dates of the parental strains are 11th December 2011 and 30th December 2011, whereas the collection date of the daughter strain is given as 6th November 2011, several weeks before the parental dates. This is not as problematic as it seems: There are ample instances of the exact same strains turning up years later (see e.g., cases cited in [10, 16, 39]), so it is possible that the parental strains our algorithm identified on the basis of strict sequence identity had actually existed earlier and their later appearance were the ones that were captured in the NCBI and our databases. There are some other matters of interest: While in the majority of instances the parental and daughter strains are found in the same general locality, there are several examples of non-obvious pairing and offspring. Entry 10 in the list of recombinants for H1N1 shows that the parents were from Kowloon and Guangdong whereas the daughter was identified from an isolate in Singapore. We had seen by our analysis earlier [16] that identical strains of neuraminidase could be found in locations thousands of kilometers apart, e.g., in Qinghai lake in China and in Astrakhan in Azerbaijan. Recent research has shown that virus persistence in the environment can be for a year or more [39]. It is not surprising therefore that related strains are found in different localities; what is surprising is that there were so few of such examples that came out in our analysis.

Additional file 1 shows another interesting fact: All valid recombinant possibilities as per our criterion were restricted to parents and daughters from the same hemagglutinin subtype, i.e. there were no examples of mixed subtype marriages.

The incidence of recombination-like events was not the same for all subtypes: There were wide variations in the percentage of recombinants or recombinant-likes discovered by our methodology—from 3.74 % for H1N1 (16 recombinants out of 427 strains), 5.71 % (20 out of 350) for H5N1, 7.35 % (30 out of 408) for H3N2 to 7.87 % (seven out of 89) for H7N9 in our database. In particular, the H3N2 with 30 recombinant-like strains out of 408 strains in our database, has 1.9 times more daughter strains than of the H1N1 varieties (16/427) although the total strains investigated are almost the same: 408 for the H3N2 and 427 (1.046 times of the H3N2) for the H1N1 (Table 1). Even H5N1 with 350 entries showed 20 daughter strains, more than what H1N1 had to offer. This seems to be in keeping with the findings that H1 and H3 subtypes of hemagglutinin have different evolutionary histories [3, 10, 28, 49].

Continuing with our observations briefly for the two other subtypes, viz., H5N1 and H7N9, we found:

H5N1—This subtype of the influenza virus attracted attention since 1997 when it infected humans in Hong Kong and was identified as a highly pathogenic avian influenza virus (HPAIV) killing or leading to culling of millions of poultry worldwide while causing 440 human fatalities in the period 2003–2015 (up to 31 March 2015) at a mortality rate of 1:2 [47]. In the period under our consideration, 20 parent–parent-daughter triplets were identified from Bangladesh to Japan. The members of each triplet are not necessarily from the same species, but generally are identified with the same country. We note in passing that no such triplets were found from China specific strains where the H5N1 HPAIV is presumed to have originated.

H7N9—This subtype was considered a low pathogenicity avian influenza virus (LPAIV) until early 2013 when it mutated to a HPAIV virus which also led to human infections with a mortality rate of about 1:3 [45]. In the period under consideration for our research, we found 7 recombination-like events in the 89 sequences we collected from GenBank. Most of these were related to avian isolates with parents and daughters in same locality or neighborhood. Interestingly, two strains of the H7N9, A/pigeon/Shanghai/S1423/2013(H7N9) and A/homing pigeon/Jiangsu/SD184/2013(H7N9), from nearby localities, both having identical HA2 segment sequences but non-identical HA1, can be considered to have paired with A/environment/Shanghai/S1438/2013(H7N9) to produce a daughter sequence—A/environment/Shanghai/S1436/2013(H7N9)—that has the HA1 identical with the Shanghai/S1438 strain but HA2 identical to both the *pigeon* strains. Two human strains from Nanjing and Suzhou, two cities relatively close by, appear to have produced a daughter strain that was collected from the environment, perhaps from effluent discharge as has been seen elsewhere [46]. Similarly, two strains from Wuxi and Changsha, slightly farther apart, also produced a daughter strain isolated from the environment.

More generally, given our hypothesis that template switching at the HA1–HA2 junction creates opportunities for recombination-like events, we have seen that HA1 from one parental viral strain and HA2 from another make up a daughter strain. It should be possible also for a daughter strain to arise from the other pairing of HA1 and HA2, i.e. one with a HA2 from the first parent and a HA1 from the second. This is seen in triplet numbers 12 and 16 of H3N2 subtype in Additional file 1: Parents A/Delhi/1183/2013(H3N2) and A/Delhi/567/2013(H3N2) gave rise to two daughter strains—A/Haryana/706/2013(H3N2) and A/Haryana/707/2013(H3N2)—with their two segments reversed. Similarly, there are instances where the same parent has paired with different strains to spawn several daughter strains: Triplet numbers 10 and 11 of H5N1 strains from Bangladesh are one example set. We have found in other instances several strains of a subtype that have identical sequence for one segment but with differences in the other segment; e.g., there are 44 strains of H7N9 in our database that have exactly the same HA2 sequence as in, say, A/Zhenjiang/1/2013(H7N9), and a group of nine strains with identical HA1 as in A/Wuxi/3/2013(H7N9). Such degeneracies are evident in the other subtypes also. The point to note is that it is possible therefore to have several different strains leading through this recombinant-like process to produce daughter viral strains that will be identical to one another in both segments. This can be seen in triplet numbers 16 and 20 of H5N1 subtype in Additional file 1 where parents A/chicken/Miyazaki/TA3/2011(H5N1) and A/chicken/Miyazaki/11/2011(H5N1) produce daughter strain A/chicken/Miyazaki/M6/2011(H5N1) which is identical in its entirety to strain A/peregrine falcon/Miyazaki/22M684/2011(H5N1) that apparently arose from parents A/peregrine falcon/Miyazaki/22M771/2011(H5N1) and A/chicken/Miyazaki/10/2011(H5N1) which are different from the previous set of parents. There is another problem that may arise in these circumstances: one parent could be considered to have paired with one of several other strains to yield one daughter strain that has the HA1 (or HA2) from the original parent but HA2 (or HA1) from any of the other parental strains; unique identification of both parental strains becomes impossible. Since the parent–parent-daughter designations are purely for convenience, we can consider whether the daughter strain could take the role of a parent also. Such a combination can be seen in triplet numbers 1 and 2 of H3N2 subtype entries in Additional file 1: one parent and daughter of triplet 1 reverse roles in triplet 2, collection dates ignored.

## Discussions

Our working hypothesis was that during viral replication it is possible that the viral polymerase that is loosely bound by only a few nucleotides to the template string [11] could jump to another equivalent template at a consensus sequence point and continue the replication process. In classical recombination explanation it is believed that in the case of break-points the replication that was interrupted by a jump to the new template proceeds to the end break-point and jumps back to the original template; this is the type of intra-segment recombination events that were investigated by Boni et al. [6]. We are looking for subunit-wise replication: Since hemagglutinin has two distinct segments, HA1 and HA2, with a consensus sequence at the junction between the two (Table 2), we had hypothesized that during replication, after reaching the end of the HA1 segment, the polymerase would jump to a different template and continue replicating, this time of the HA2, until it reaches the end of the gene sequence. For the consensus sequence, notice that the HA1-HA2 junction has a fairly conserved sequence motif: Table 2 lists the sequence motifs for 10 nucleotides around the HA1–HA2 junction for 27 sequences by way of example. Given the conserved nature of the motif, we can hypothesize that it can serve to ‘fool’ the RNA polymerase.

In general, when only one influenza strain is undergoing multiplication, such template switching may still occur, but will not produce any observable difference in the progenies. However, in the instance where more than one influenza strain infects a single cell of the host species, template switching at the segment junction can lead to progenies that contain HA1 from one parent and the HA2 from the other parent. The new combination of the HA1 and HA2 in the daughter gene may be novel, and sometimes improve the functioning of the virus.

The fact that, in the general recombination case, several daughter sequences seem to have been identified before their deemed parents were determined leads to an apparent incongruity which needs to be resolved. Our research into spread of the H5N1 influenza virus through their associated neuraminidase had shown that identical sequences had cropped up over long separations in space and time [16]; recent research estimates limits of existence of an isolated influenza virus in a wetland environment to a few days to over a year depending on salinity, pH, temperature and other factors [39]. We suggest that if the same scenario holds true for the hemagglutinin subtypes too, it is conceivable that the parents that gave rise to the identified daughter sequence had existed in the wild long enough, at least in some instances, without mutations and were identified and sequenced much later. Note that the sequences identified as parents in the recombination event identified in Boni et al. [6] were timewise separated from the daughter specimen by 4–29 years!

Our analyses above show that in about 5.95 ± 0.22 % of the cases of the 1274 strains of H1N1, H5N1, H3N2 and H7N9 from Asia in the years 2010–2014, there appears to be possibilities that template switching had taken place during replication leading to formation of daughter viral strains. Following Boni et al. [4], we then investigated whether these were isolated instances with no progenies to progress or laboratory constructs [4]. We selected a few daughter strains from each influenza subtype and subjected them to a BLASTn search to identify further examples of such strains. In each case we found several strains that appeared identical to the daughter strains or differed at most by one mutation or two. If our hypothesis of development of daughter strains by segment exchange were to be true, then this would be what we would expect to find.

Next, we compared each segment of the daughter strain with the same segment of the “wrong” parent; i.e. if HA1 of the daughter strain was inherited from the HA1 of parent P1 and HA2 from parent P2, we compared HA1 of the daughter with the HA1 of parent P2. In general, the differences were found to be of 2–3 mutations. This raises the question whether such differences could have arisen spontaneously and therefore makes the hypothesis of segment exchange unnecessary. That is possible but we had made the condition that we identify cases of template switching by considering exact matches; that we could find so many instances, viz., 73 (5.73 %) out of 1274 strains studied, makes segment exchange more probable.

The more stringent condition that daughter strains are identified after the parents, and that the parents and daughters are from the same country—preferably the same locality, also yielded several examples. It appears that these examples meet all conditions for recombination through segment exchange.

Extending our analysis to cover new complete hemagglutinin sequences uploaded in GenBank for 2015, we found nine new strains of H1N1, 89 new strains of H3N2 and four new strains of H7N9; there were no new strains of 2015 for H5N1 types as on date of accession (15th January 2016). The majority of these strains were from Japan; presumably wider geographical sourcing will be available in later periods. Computing the *g*_*R*_ values of the HA1 and HA2 segments of all the new strains to facilitate comparison of the sequences, we found that (1) none of these strains gave rise to any recombinant viruses with the strains from 2010–2014, probably due to very localized sourcing of sequences; (b) there were no recombinants between the various subtypes as we noticed in the 2010–2014 cases also; (c) there were no new recombinants in case of H1N1 and H7N9, perhaps due to very few data, but (d) we did find five instances of recombination-like events in the 89 strains of H3N2, i.e. 5.62 % of the 2015 strains of H3N2 influenza subtype showed sequence segment similarities indicative of recombination-like events that may have taken place. Given that we had found 30 instances out of 408 strains, i.e. 7.35 %, of the H3N2 of 2010–2014 period indicative of recombination-like behavior, coupled with the instances of 2015, the total comes to 35 instances our of 497 strains of H3N2, i.e. 7.04 %, showing such possible recombinations. The new recombinants are given separately in Table 3.

**Table 3:** Recombination-like events observed in complete sequences of influenza A hemagglutinin of 2015 available in Genbank

Sub type	No	Locus ID	Description	g_R_ HA1	g_R_ HA2	Date of collection
H3N2	1	KT374339	A/Japan/NHRC_GWA0186/2015(H3N2)	64.61024	37.96143	12-Jan-15
		CY193773	A/Japan/4520/2015(H3N2)	64.67959	38.35854	13-Jan-15
		CY193771	A/Japan/4518/2015(H3N2)	64.61024	38.35854	13-Jan-15
	2	KT277832	A/Japan/NHRC_GWA0184/2015(H3N2)	64.61024	38.00755	8-Jan-15
		CY193902	A/Japan/4649/2015(H3N2)	65.7185	38.35854	31-Jan-15
		CY193771	A/Japan/4518/2015(H3N2)	64.61024	38.35854	13-Jan-15
	4	CY194016	A/Japan/4763/2015(H3N2)	66.33004	39.83783	13-Feb-15
		KP877371	A/Bangkok/SI-MI31/2015(H3N2)	61.69833	38.35854	20-Feb-15
		CY194020	A/Japan/4767/2015(H3N2)	66.33004	38.35854	21-Feb-15
	4	KT220434	A/Japan/NHRC_20-N2486/2015(H3N2)	61.83264	35.34615	23-Jan-15
		CY193680	A/Japan/4427/2015(H3N2)	61.83264	34.03069	8-Jan-15
		CY193681	A/Japan/4428/2015(H3N2)	61.83264	34.03069	8-Jan-15
	5	KT220428	A/Japan/NHRC_01-C2155/2015(H3N2)	64.17716	40.44237	5-Jan-15
		CY193777	A/Japan/4524/2015(H3N2)	63.63748	39.89609	14-Jan-15
		CY193894	A/Japan/4641/2015(H3N2)	64.17716	39.89609	13-Jan-15

*Accession date* 15th January 2016

## Conclusions

Thus, we have identified 73 instances out of 1274 hemagglutinin sequences of 2010–2014, and five more out of new 102 strains of 2015, where recombination-like events within the restricted scenario of subunit segment exchange seem to have taken place. Intra-segment recombination through exchanges of small sections of nucleotide sequences through break-point mechanisms appear controversial; Boni et al. [6] had estimated that such recombination, if they existed, could not exceed 2 % of the cases they investigated, whereas, in contrast, our finding shows over 5 % cases of whole segment exchange. Perhaps there are many more processes going on in influenza replication than have been conceived of so far.

## Availability of supporting data

Data used in this research are freely available and have been downloaded from National Institute of Health GenBank database (http://www.ncbi.nlm.nih.gov/). For completeness, the sequences showing evidence of segment exchange recombination, and listed in Additional file 1 and Table 3, are given separately in Additional file 2.

## Additional files

10.1186/s13104-016-2017-3 Parent–Parent-Daughter sequences identified by our algorithm from the database of H1N1, H5N1, H3N2 and H7N9 hemagglutinin sequences. In each triplet the first entry is the HA1 donor, the second entry is the HA2 donor and the third entry is the daughter sequence.
10.1186/s13104-016-2017-3 Complete sequence data of the hemagglutinins identified as leading to recombination-like events listed in Additional file 1 and Table 3 are included as text files in the compressed file SEQUENCES.ZIP.

## Abbreviations

LPAIV: low pathogenicity avian influenza virus
HPAIV: highly pathogenic avian influenza virus
WHO: World Health Organization
