# Supplementary material for: Bioinformatics studies of Influenza A hemagglutinin sequence data indicate recombination-like events leading to segment exchanges
Source: BMC Res Notes. 2016 Apr 15;9:222. doi: 10.1186/s13104-016-2017-3 (PMC4832483; doi:10.1186/s13104-016-2017-3)
Supplement: Supplementary file 1 — 10.1186/s13104-016-2017-3 Parent–Parent-Daughter sequences identified by our algorithm from the database of H1N1, H5N1, H3N2 and H7N9 hemagglutinin sequences. In each triplet the first entry is the HA1 donor, the second entry is the HA2 donor and the third entry is the daughter sequence. [file 13104_2016_2017_MOESM1_ESM.docx]

**Table 3 (Additional table):** Parent-Parent-Daughter sequences identified by our algorithm from the database of H1N1, H5N1, H3N2 and H7N9 hemagglutinin sequences. In each triplet the first entry is the HA1 donor, the second entry is the HA2 donor and the third entry is the daughter sequence

| **Sub Type** | **No** | **Locus ID** | **Description** | **g_R_ HA1** | **g_R_ HA2** | **Date of collection** |
| --- | --- | --- | --- | --- | --- | --- |
|  |  |  |  |  |  |  |
| **H1N1** | 1 | KM029055 | A/swine/Hong Kong/NS4846/2011(H1N1) | 66.18646 | 49.33498 | 15-Dec-11 |
|  |  | KM029103 | A/swine/Hong Kong/4902/2011(H1N1) | 65.75885 | 49.06597 | 15-Dec-11 |
|  |  | KM029063 | A/swine/Hong Kong/NS4848/2011(H1N1) | 66.18646 | 49.06597 | 15-Dec-11 |
|  |  |  |  |  |  |  |
|  | 2 | KM028335 | A/swine/Guangxi/3614/2011(H1N1) | 67.93622 | 49.80615 | 11-Dec-11 |
|  |  | KM028423 | A/swine/Guangxi/3880/2011(H1N1) | 69.77442 | 48.80638 | 30-Dec-11 |
|  |  | KM028271 | A/swine/Guangxi/NS3248/2011(H1N1) | 67.93622 | 48.80638 | 6-Nov-11 |
|  |  |  |  |  |  |  |
|  | 3 | JX309126 | A/Singapore/KK12/2010(H1N1) | 70.02491 | 41.09317 | 6-Jan-10 |
|  |  | JX309106 | A/Singapore/GP205/2010(H1N1) | 68.65045 | 41.02775 | 13-Jan-10 |
|  |  | JX309009 | A/Singapore/GP241/2010(H1N1) | 70.02491 | 41.02775 | 15-Jan-10 |
|  |  |  |  |  |  |  |
|  | 4 | CY124761 | A/Singapore/TT117/2011(H1N1) | 70.06969 | 40.13403 | 7-Feb-11 |
|  |  | CY124694 | A/Singapore/GP924/2011(H1N1) | 70.16010 | 39.36419 | 8-Apr-11 |
|  |  | CY124765 | A/Singapore/TT127/2011(H1N1) | 70.06969 | 39.36419 | 10-Feb-11 |
|  |  |  |  |  |  |  |
|  | 5 | AB898078 | A/Yamagata/87/2013(H1N1) | 70.46402 | 42.78919 | 2/8/2013 |
|  |  | CY163409 | A/Japan/3421/2013(H1N1) | 69.15273 | 44.21400 | 17-Feb-13 |
|  |  | CY163415 | A/Japan/3427/2013(H1N1) | 70.46402 | 44.21400 | 20-Feb-13 |
|  |  |  |  |  |  |  |
|  | 6 | JN381321 | A/Taiwan/90157/2011(H1N1) | 70.64320 | 39.78396 | Feb-11 |
|  |  | JQ693691 | A/Taiwan/1829/2011(H1N1) | 71.08991 | 38.45775 | 24-Feb-11 |
|  |  | JQ693693 | A/Taiwan/4696/2011(H1N1) | 70.64320 | 38.45775 | 22-Feb-11 |
|  |  |  |  |  |  |  |
|  | 7 | KC190063 | A/swine/Colombo/N57/2011(H1N1) | 71.00280 | 41.40772 | 17-Feb-11 |
|  |  | KC190062 | A/swine/Colombo/N50/2011(H1N1) | 69.81966 | 41.80332 | 8-Feb-11 |
|  |  | KC190055 | A/swine/Colombo/N62/2011(H1N1) | 71.00280 | 41.80332 | 17-Feb-11 |
|  |  |  |  |  |  |  |
|  | 8 | CY187688 | A/Korea/3770/2014(H1N1) | 71.07904 | 38.68499 | 28-Jan-14 |
|  |  | CY187703 | A/Korea/3785/2014(H1N1) | 72.02395 | 39.07675 | 13-Mar-14 |
|  |  | KJ946415 | A/Shanghai/Mix1/2014(H1N1) | 71.07904 | 39.07675 | 3-Jan-14 |
|  |  |  |  |  |  |  |
|  | 9 | CY124713 | A/Singapore/KK157/2011(H1N1) | 71.53705 | 41.88033 | 28-Mar-11 |
|  |  | CY091702 | A/Singapore/KK105/2011(H1N1) | 70.98168 | 41.08333 | 11-Feb-11 |
|  |  | CY124544 | A/Singapore/GP414/2011(H1N1) | 71.53705 | 41.08333 | 1-Feb-11 |
|  |  |  |  |  |  |  |
|  | 10 | CY129582 | A/Kowloon/INS531/2011(H1N1) | 71.56326 | 40.38101 | 15-Feb-11 |
|  |  | JF929771 | A/Guangdong/029/2011(H1N1) | 72.12275 | 40.25289 | 21-Feb-11 |
|  |  | CY124776 | A/Singapore/TT157/2011(H1N1) | 71.56326 | 40.25289 | 23-Feb-11 |
|  |  |  |  |  |  |  |
|  | 11 | AB745363 | A/Tottori/TT114/2011(H1N1) | 72.02846 | 41.69012 | Feb-11 |
|  |  | AB745408 | A/Tottori/YK058/2011(H1N1) | 68.79656 | 41.80737 | Feb-11 |
|  |  | AB745378 | A/Tottori/TT254/2011(H1N1) | 72.02846 | 41.80737 | Apr-11 |
|  |  |  |  |  |  |  |
|  | 12 | AB745369 | A/Tottori/TT150/2011(H1N1) | 74.10208 | 41.70472 | Mar-11 |
|  |  | AB745373 | A/Tottori/TT183/2011(H1N1) | 74.94230 | 42.17455 | Mar-11 |
|  |  | AB745367 | A/Tottori/TT141/2011(H1N1) | 74.10208 | 42.17455 | Feb-11 |
|  |  |  |  |  |  |  |
|  | 13 | KM027719 | A/swine/Guangdong/NS3714/2011(H1N1) | 74.35366 | 44.16740 | 10-Nov-11 |
|  |  | KM027743 | A/swine/Guangdong/3751/2011(H1N1) | 74.72778 | 45.20284 | 10-Nov-11 |
|  |  | KM027671 | A/swine/Guangdong/NS3694/2011(H1N1) | 74.35366 | 45.20284 | 10-Nov-11 |
|  |  |  |  |  |  |  |
|  | 14 | AB745370 | A/Tottori/TT151/2011(H1N1) | 74.83906 | 42.20102 | Mar-11 |
|  |  | AB745364 | A/Tottori/TT126/2011(H1N1) | 74.49133 | 42.17455 | Feb-11 |
|  |  | AB745326 | A/Tottori/ST740/2011(H1N1) | 74.83906 | 42.17455 | Apr-11 |
|  |  |  |  |  |  |  |
|  | 15 | KM027775 | A/swine/Guangdong/NS94/2012(H1N1) | 76.05366 | 45.12493 | 11-Jan-12 |
|  |  | KM027783 | A/swine/Guangdong/NS99/2012(H1N1) | 75.92379 | 45.21387 | 11-Jan-12 |
|  |  | KM027767 | A/swine/Guangdong/NS91/2012(H1N1) | 76.05366 | 45.21387 | 11-Jan-12 |
|  |  |  |  |  |  |  |
|  | 16 | JN381321 | A/Taiwan/90157/2011(H1N1) | 70.64320 | 39.78396 | Feb-11 |
|  |  | JN381340 | A/Taiwan/65569/2011(H1N1) | 71.62651 | 38.45775 | Feb-11 |
|  |  | JQ693695 | A/Taiwan/4711/2011(H1N1) | 70.64320 | 38.45775 | 14-Mar-11 |
|  |  |  |  |  |  |  |
|  |  |  |  |  |  |  |
| **H3N2** | 1 | KM069501 | A/Singapore/C2010.307/2010(H3N2) | 57.06303 | 36.58159 | 22-Apr-10 |
|  |  | JX437712 | A/Singapore/C2010.036V/2010(H3N2) | 59.02475 | 37.64934 | 13-Jan-10 |
|  |  | JX437832 | A/Singapore/H2010.370C/2010(H3N2) | 57.06303 | 37.64934 | 15-May-10 |
|  |  |  |  |  |  |  |
|  | 2 | JX437832 | A/Singapore/H2010.370C/2010(H3N2) | 57.06303 | 37.64934 | 15-May-10 |
|  |  | KM069503 | A/Singapore/H2010.310/2010(H3N2) | 56.53434 | 36.58159 | 23-Apr-10 |
|  |  | KM069501 | A/Singapore/C2010.307/2010(H3N2) | 57.06303 | 36.58159 | 22-Apr-10 |
|  |  |  |  |  |  |  |
|  | 3 | CY124157 | A/Singapore/EN358/2011(H3N2) | 58.75677 | 34.62476 | 9-Sep-11 |
|  |  | CY116638 | A/Tbilisi/GNCDC0557/2012(H3N2) | 58.71523 | 36.24932 | 28-Mar-12 |
|  |  | KF014137 | A/Singapore/C2011.452/2011(H3N2) | 58.75677 | 36.24932 | 14-Jun-11 |
|  |  |  |  |  |  |  |
|  | 4 | CY100091 | A/Singapore/GP3268/2010(H3N2) | 59.02475 | 37.58014 | 24-Jun-10 |
|  |  | JX437714 | A/Singapore/C2010.362V/2010(H3N2) | 59.39212 | 37.64934 | 12-May-10 |
|  |  | JX437712 | A/Singapore/C2010.036V/2010(H3N2) | 59.02475 | 37.64934 | 13-Jan-10 |
|  |  |  |  |  |  |  |
|  | 5 | CY124165 | A/Singapore/GP103/2011(H3N2) | 59.30521 | 36.35063 | 12-Jan-11 |
|  |  | JX437719 | A/Singapore/C2011.027V/2011(H3N2) | 59.32785 | 34.82836 | 10-Jan-11 |
|  |  | CY124303 | A/Singapore/KK114/2011(H3N2) | 59.30521 | 34.82836 | 16-Feb-11 |
|  |  |  |  |  |  |  |
|  | 6 | KF952378 | A/Delhi/1702/2013(H3N2) | 59.35908 | 37.83930 | 9-Feb-13 |
|  |  | KF952380 | A/Delhi/1782/2013(H3N2) | 59.64999 | 36.77863 | 20-Feb-13 |
|  |  | KF952379 | A/Delhi/1720/2013(H3N2) | 59.35908 | 36.77863 | 20-Feb-13 |
|  |  |  |  |  |  |  |
|  | 7 | CY147300 | A/Japan/3408/2013(H3N2) | 59.64999 | 38.26390 | 8-Mar-13 |
|  |  | KM208530 | A/Hangzhou/B48/2013(H3N2) | 63.06078 | 37.97303 | 29-Jan-13 |
|  |  | CY147303 | A/Japan/3411/2013(H3N2) | 59.64999 | 37.97303 | 26-Mar-13 |
|  |  |  |  |  |  |  |
|  | 8 | KM208559 | A/Hangzhou/B721/2013(H3N2) | 59.64999 | 35.56911 | 25-Nov-13 |
|  |  | KM208549 | A/Hangzhou/A773/2013(H3N2) | 61.43477 | 35.75732 | 15-Nov-13 |
|  |  | KM208540 | A/Hangzhou/A649/2013(H3N2) | 59.64999 | 35.75732 | 10-Oct-13 |
|  |  |  |  |  |  |  |
|  | 9 | KM208533 | A/Hangzhou/A155/2013(H3N2) | 59.64999 | 37.59167 | 25-Feb-13 |
|  |  | KM208568 | A/Hangzhou/B750/2013(H3N2) | 59.67293 | 36.15799 | 5-Dec-13 |
|  |  | KM208544 | A/Hangzhou/B599/2013(H3N2) | 59.64999 | 36.15799 | 15-Oct-13 |
|  |  |  |  |  |  |  |
|  | 10 | KM208544 | A/Hangzhou/B599/2013(H3N2) | 59.64999 | 36.15799 | 15-Oct-13 |
|  |  | KJ577189 | A/Thailand/VIROAF6/2012(H3N2) | 58.98829 | 36.42732 | 13-Sep-12 |
|  |  | KM208522 | A/Hangzhou/A289/2012(H3N2) | 59.64999 | 36.42732 | 13-Jul-12 |
|  |  |  |  |  |  |  |
|  | 11 | KM208551 | A/Hangzhou/A776/2013(H3N2) | 59.70448 | 39.19308 | 18-Nov-13 |
|  |  | KM208562 | A/Hangzhou/B728/2013(H3N2) | 60.50717 | 39.09160 | 27-Nov-13 |
|  |  | KM208556 | A/Hangzhou/A796/2013(H3N2) | 59.70448 | 39.09160 | 25-Nov-13 |
|  |  |  |  |  |  |  |
|  | 12 | KF014183 | A/Singapore/H2011.479/2011(H3N2) | 59.83328 | 35.14717 | 24-Jun-11 |
|  |  | KF014185 | A/Singapore/H2011.496/2011(H3N2) | 60.04087 | 35.56922 | 30-Jun-11 |
|  |  | KF014138 | A/Singapore/C2011.458/2011(H3N2) | 59.83328 | 35.56922 | 16-Jun-11 |
|  |  |  |  |  |  |  |
|  | 13 | CY124315 | A/Singapore/KK200/2011(H3N2) | 59.99304 | 36.29336 | 14-Jun-11 |
|  |  | CY124225 | A/Singapore/GP1507/2011(H3N2) | 60.00438 | 35.56922 | 13-Sep-11 |
|  |  | KF014136 | A/Singapore/C2011.422/2011(H3N2) | 59.99304 | 35.56922 | 3-Jun-11 |
|  |  |  |  |  |  |  |
|  | 14 | CY124379 | A/Singapore/MOH12/2011(H3N2) | 59.99304 | 35.64061 | 20-Jun-11 |
|  |  | CY124163 | A/Singapore/EN514/2011(H3N2) | 57.70854 | 36.24932 | 7-Dec-11 |
|  |  | CY124389 | A/Singapore/TT203/2011(H3N2) | 59.99304 | 36.24932 | 23-May-11 |
|  |  |  |  |  |  |  |
|  | 15 | KM028487 | A/swine/Guangxi/NS1409/2012(H3N2) | 60.64424 | 37.49119 | 3-Jun-12 |
|  |  | KM029439 | A/swine/Hong Kong/NS2168/2012(H3N2) | 60.65371 | 37.32557 | 21-Jun-12 |
|  |  | KM028479 | A/swine/Guangxi/NS1402/2012(H3N2) | 60.64424 | 37.32557 | 3-Jun-12 |
|  |  |  |  |  |  |  |
|  | 16 | CY124205 | A/Singapore/GP1374/2011(H3N2) | 61.36125 | 33.97630 | 2-Aug-11 |
|  |  | KF014185 | A/Singapore/H2011.496/2011(H3N2) | 60.04087 | 35.56922 | 30-Jun-11 |
|  |  | CY124197 | A/Singapore/GP1297/2011(H3N2) | 61.36125 | 35.56922 | 19-Jul-11 |
|  |  |  |  |  |  |  |
|  | 17 | CY099953 | A/Guangdong/94/2011(H3N2) | 62.50542 | 34.28743 | 9-Mar-11 |
|  |  | KF014131 | A/Singapore/C2011.173/2011(H3N2) | 63.36273 | 34.49244 | 4-Mar-11 |
|  |  | CY124297 | A/Singapore/GP5/2011(H3N2) | 62.50542 | 34.49244 | 4-Jan-11 |
|  |  |  |  |  |  |  |
|  | 18 | KF952368 | A/Delhi/1183/2013(H3N2) | 62.63678 | 35.72909 | 16-Feb-13 |
|  |  | KF952364 | A/Delhi/567/2013(H3N2) | 62.69224 | 36.77863 | 12-Feb-13 |
|  |  | KF952365 | A/Haryana/706/2013(H3N2) | 62.63678 | 36.77863 | 13-Feb-13 |
|  |  |  |  |  |  |  |
|  | 19 | KM069585 | A/Singapore/H2013.422a/2013(H3N2) | 62.64228 | 35.67439 | 3-Jun-13 |
|  |  | KM069595 | A/Singapore/H2013.696/2013(H3N2) | 64.41480 | 38.02926 | 11-Sep-13 |
|  |  | KM069586 | A/Singapore/H2013.422b/2013(H3N2) | 62.64228 | 38.02926 | 3-Jun-13 |
|  |  |  |  |  |  |  |
|  | 20 | CY147294 | A/Korea/3402/2013(H3N2) | 62.65824 | 37.51082 | 20-Feb-13 |
|  |  | CY187678 | A/Japan/3760/2014(H3N2) | 63.55433 | 36.72179 | 5-Feb-14 |
|  |  | CY147302 | A/Korea/3410/2013(H3N2) | 62.65824 | 36.72179 | 16-Mar-13 |
|  |  |  |  |  |  |  |
|  | 21 | KF952385 | A/Haryana/2396/2013(H3N2) | 62.68156 | 36.91641 | 24-Feb-13 |
|  |  | KF952369 | A/Delhi/1191/2013(H3N2) | 62.28075 | 38.65581 | 16-Feb-13 |
|  |  | KF952370 | A/Delhi/1254/2013(H3N2) | 62.68156 | 38.65581 | 16-Feb-13 |
|  |  |  |  |  |  |  |
|  | 22 | KF952364 | A/Delhi/567/2013(H3N2) | 62.69224 | 36.77863 | 12-Feb-13 |
|  |  | KF952368 | A/Delhi/1183/2013(H3N2) | 62.63678 | 35.72909 | 16-Feb-13 |
|  |  | KF952366 | A/Haryana/707/2013(H3N2) | 62.69224 | 35.72909 | 13-Feb-13 |
|  |  |  |  |  |  |  |
|  | 23 | KF952366 | A/Haryana/707/2013(H3N2) | 62.69224 | 35.72909 | 13-Feb-13 |
|  |  | KF952379 | A/Delhi/1720/2013(H3N2) | 59.35908 | 36.77863 | 20-Feb-13 |
|  |  | KF952364 | A/Delhi/567/2013(H3N2) | 62.69224 | 36.77863 | 12-Feb-13 |
|  |  |  |  |  |  |  |
|  | 24 | CY124221 | A/Singapore/GP1490/2011(H3N2) | 62.81143 | 36.67174 | 8-Sep-11 |
|  |  | CY124223 | A/Singapore/GP1503/2011(H3N2) | 62.96305 | 36.70441 | 13-Sep-11 |
|  |  | KC020359 | A/Cambodia/V1005380/2011(H3N2) | 62.81143 | 36.70441 | 3-Oct-11 |
|  |  |  |  |  |  |  |
|  | 25 | KF952377 | A/Delhi/1553/2013(H3N2) | 62.93095 | 36.25626 | 19-Feb-13 |
|  |  | KF952376 | A/Delhi/1550/2013(H3N2) | 62.21697 | 37.31152 | 19-Feb-13 |
|  |  | KF952375 | A/Delhi/1549/2013(H3N2) | 62.93095 | 37.31152 | 19-Feb-13 |
|  |  |  |  |  |  |  |
|  | 26 | KF952377 | A/Delhi/1553/2013(H3N2) | 62.93095 | 36.25626 | 19-Feb-13 |
|  |  | KF952365 | A/Haryana/706/2013(H3N2) | 62.63678 | 36.77863 | 13-Feb-13 |
|  |  | KF952367 | A/Delhi/764/2013(H3N2) | 62.93095 | 36.77863 | 13-Feb-13 |
|  |  |  |  |  |  |  |
|  | 27 | CY124401 | A/Singapore/TT241/2011(H3N2) | 62.93209 | 35.77063 | 27-Jun-11 |
|  |  | KF952358 | A/Goa/8773/2011(H3N2) | 62.65420 | 35.74676 | 7-Jan-11 |
|  |  | KF952357 | A/Delhi/8865/2011(H3N2) | 62.93209 | 35.74676 | 7-Feb-11 |
|  |  |  |  |  |  |  |
|  | 28 | KF952386 | A/Delhi/2487/2013(H3N2) | 62.97570 | 35.85724 | 25-Feb-13 |
|  |  | KF952385 | A/Haryana/2396/2013(H3N2) | 62.68156 | 36.91641 | 24-Feb-13 |
|  |  | KF952384 | A/Delhi/2277/2013(H3N2) | 62.97570 | 36.91641 | 23-Feb-13 |
|  |  |  |  |  |  |  |
|  | 29 | KM069579 | A/Singapore/H2012.934/2012(H3N2) | 62.98526 | 38.37652 | 7-Dec-12 |
|  |  | KF432083 | A/Singapore/H2013.060/2013(H3N2) | 63.07703 | 36.27213 | 22-Jan-13 |
|  |  | KM069574 | A/Singapore/C2012.801/2012(H3N2) | 62.98526 | 36.27213 | 19-Oct-12 |
|  |  |  |  |  |  |  |
|  | 30 | KF014135 | A/Singapore/C2011.411/2011(H3N2) | 64.48454 | 35.78454 | 30-May-11 |
|  |  | CY124207 | A/Singapore/GP1382/2011(H3N2) | 60.33210 | 35.77063 | 3-Aug-11 |
|  |  | CY124173 | A/Singapore/GP1072/2011(H3N2) | 64.48454 | 35.77063 | 6-Jun-11 |
|  |  |  |  |  |  |  |
|  |  |  |  |  |  |  |
| **H5N1** | 1 | KF369229 | A/chicken/Cambodia/W0530391/2012(H5N1) | 61.16050 | 37.63436 | 28-May-12 |
|  |  | KF369214 | A/Cambodia/W0526301/2012(H5N1) | 60.92035 | 37.15533 | 25-May-12 |
|  |  | KF369222 | A/chicken/Cambodia/W0530389/2012(H5N1) | 61.16050 | 37.15533 | 28-May-12 |
|  |  |  |  |  |  |  |
|  | 2 | KF001474 | A/civet cat/Cambodia/X0313306/2013(H5N1) | 61.85977 | 36.95179 | 13-Mar-13 |
|  |  | KF001497 | A/duck/Cambodia/X0220302/2013(H5N1) | 60.53024 | 39.06615 | 18-Feb-13 |
|  |  | KF001478 | A/civet cat/Cambodia/X0313307/2013(H5N1) | 61.85977 | 39.06615 | 13-Mar-13 |
|  |  |  |  |  |  |  |
|  | 3 | JN588821 | A/duck/Cambodia/PV027D1/2010(H5N1) | 63.43130 | 32.76611 | 30-Apr-10 |
|  |  | JN588819 | A/duck/Cambodia/TK007D5T/2010(H5N1) | 64.58104 | 34.57046 | 10-Feb-10 |
|  |  | JN588813 | A/duck/Cambodia/TK05D8T/2010(H5N1) | 63.43130 | 34.57046 | 12-Feb-10 |
|  |  |  |  |  |  |  |
|  | 4 | CY098304 | A/chicken/Lao/LH2/2010(H5N1) | 65.19016 | 33.04659 | May-10 |
|  |  | CY098358 | A/duck/Lao/567/2010(H5N1) | 65.60155 | 34.50061 | Mar-10 |
|  |  | CY098297 | A/chicken/Lao/LH1/2010(H5N1) | 65.19016 | 34.50061 | May-10 |
|  |  |  |  |  |  |  |
|  | 5 | JQ714220 | A/Cambodia/V0813302/2011(H5N1) | 67.12363 | 37.07005 | 18-Aug-11 |
|  |  | JQ714246 | A/Cambodia/W0112303/2012(H5N1) | 67.54300 | 36.95355 | 10-Jan-12 |
|  |  | JQ701712 | A/marabou stork/Cambodia/TM068/2011(H5N1) | 67.12363 | 36.95355 | 14-Jul-11 |
|  |  |  |  |  |  |  |
|  | 6 | KP097914 | A/muscovy duck/Vietnam/NCVD-KA395/2012(H5N1) | 67.12859 | 37.43660 | 15-Oct-12 |
|  |  | AB780486 | A/duck/Vietnam/OIE-2236/2012(H5N1) | 67.33976 | 35.63658 | 11-Aug-12 |
|  |  | AB786676 | A/muscovy duck/Vietnam/LBM227/2012(H5N1) | 67.12859 | 35.63658 | 2012 |
|  |  |  |  |  |  |  |
|  | 7 | KM821610 | A/duck/Khanhhoa/CVVI-05/2013(H5N1) | 67.87082 | 37.13120 | 7-Jan-13 |
|  |  | KM821607 | A/chicken/Khanhhoa/CVVI-02/2013(H5N1) | 69.67067 | 37.43660 | 2-Jan-13 |
|  |  | KM821606 | A/duck/Khanhhoa/CVVI-01/2013(H5N1) | 67.87082 | 37.43660 | 2-Jan-13 |
|  |  |  |  |  |  |  |
|  | 8 | AB684246 | A/chicken/Miyazaki/N7/2011(H5N1) | 68.12188 | 37.53153 | Jan-11 |
|  |  | AB747155 | A/mandarin duck/Miyazaki/22M-765/2011(H5N1) | 71.05870 | 37.03830 | 2/14/2011 |
|  |  | AB747161 | A/peregrine falcon/Miyazaki/22M771/2011(H5N1) | 68.12188 | 37.03830 | 15-Feb-11 |
|  |  |  |  |  |  |  |
|  | 9 | AB615237 | A/tufted duck/Fukushima/2/2011(H5N1) | 68.44021 | 36.67017 | Jan-11 |
|  |  | AB629698 | A/tufted duck/Fukushima/16/2011(H5N1) | 68.52665 | 35.95942 | Jan-11 |
|  |  | AB675536 | A/tufted duck/Fukushima/5/2011(H5N1) | 68.44021 | 35.95942 | Jan-11 |
|  |  |  |  |  |  |  |
|  | 10 | CY066012 | A/chicken/Bhutan/248007/2010(H5N1) | 68.48216 | 39.13266 | 17-Feb-10 |
|  |  | CY063481 | A/chicken/Bhutan/248015/2010(H5N1) | 68.70911 | 39.39331 | 19-Feb-10 |
|  |  | HQ156776 | A/chicken/Bangladesh/1151-11/2010(H5N1) | 68.48216 | 39.39331 | 2010 |
|  |  |  |  |  |  |  |
|  | 11 | AB677867 | A/mandarin duck/Nagasaki/4202A023/2011(H5N1) | 68.55248 | 37.31454 | 4-Feb-11 |
|  |  | AB747107 | A/mandarin duck/Nagasaki/4201A012/2011(H5N1) | 68.32758 | 37.03830 | 31-Jan-11 |
|  |  | AB747138 | A/mandarin duck/Oita/4402F038/2011(H5N1) | 68.55248 | 37.03830 | 7-Feb-11 |
|  |  |  |  |  |  |  |
|  | 12 | KF182741 | A/duck/Vietnam/QB1207/2012(H5N1) | 68.67336 | 36.29922 | 28-Jul-12 |
|  |  | AB780478 | A/duck/Vietnam/OIE-2211/2012(H5N1) | 68.66040 | 39.67092 | 11-Aug-12 |
|  |  | AB769252 | A/duck/Vietnam/OIE-2202/2012(H5N1) | 68.67336 | 39.67092 | 11-Aug-12 |
|  |  |  |  |  |  |  |
|  | 13 | AB684259 | A/chicken/Nara/1/2011(H5N1) | 68.87510 | 37.97111 | Feb-11 |
|  |  | AB684255 | A/chicken/Aichi/2/2011(H5N1) | 69.83166 | 37.03830 | Feb-11 |
|  |  | AB684256 | A/chicken/Wakayama/1/2011(H5N1) | 68.87510 | 37.03830 | Feb-11 |
|  |  |  |  |  |  |  |
|  | 14 | AB747167 | A/black headed gull/Tottori/1-009/2011(H5N1) | 69.64143 | 37.06276 | 19-Jan-11 |
|  |  | AB747114 | A/common pochard/Tottori/3102S012/2011(H5N1) | 68.42295 | 36.85819 | 3-Feb-11 |
|  |  | AB747169 | A/tufted duck/Tottori/1-012/2011(H5N1) | 69.64143 | 36.85819 | 24-Jan-11 |
|  |  |  |  |  |  |  |
|  | 15 | AB747169 | A/tufted duck/Tottori/1-012/2011(H5N1) | 69.64143 | 36.85819 | 24-Jan-11 |
|  |  | AB747111 | A/tufted duck/Tottori/3102S004/2011(H5N1) | 69.62215 | 37.06276 | 1-Feb-11 |
|  |  | AB747167 | A/black headed gull/Tottori/1-009/2011(H5N1) | 69.64143 | 37.06276 | 19-Jan-11 |
|  |  |  |  |  |  |  |
|  | 16 | AB684247 | A/chicken/Miyazaki/TA3/2011(H5N1) | 69.85153 | 36.89113 | Jan-11 |
|  |  | AB684253 | A/chicken/Miyazaki/11/2011(H5N1) | 68.37027 | 37.03830 | Feb-11 |
|  |  | AB675739 | A/chicken/Miyazaki/M6/2011(H5N1) | 69.85153 | 37.03830 | Jan-11 |
|  |  |  |  |  |  |  |
|  | 17 | KJ682250 | A/chicken/Bhutan/406/2012(H5N1) | 72.71906 | 32.18860 | 16-Jan-12 |
|  |  | KJ682226 | A/chicken/Bhutan/415/2012(H5N1) | 72.83374 | 34.91408 | 18-Feb-12 |
|  |  | KJ682260 | A/chicken/Bhutan/413/2012(H5N1) | 72.71906 | 34.91408 | 18-Feb-12 |
|  |  |  |  |  |  |  |
|  | 18 | KF888486 | A/environment/Bangladesh/15147/2012(H5N1) | 74.49882 | 30.49544 | 18-Jan-12 |
|  |  | KF888400 | A/environment/Bangladesh/15126/2012(H5N1) | 74.31136 | 31.00304 | 18-Jan-12 |
|  |  | KF888580 | A/environment/Bangladesh/15145/2012(H5N1) | 74.49882 | 31.00304 | 18-Jan-12 |
|  |  |  |  |  |  |  |
|  | 19 | KF888432 | A/environment/Bangladesh/15114/2012(H5N1) | 74.53939 | 30.49544 | 18-Jan-12 |
|  |  | KF888400 | A/environment/Bangladesh/15126/2012(H5N1) | 74.31136 | 31.00304 | 18-Jan-12 |
|  |  | KF888554 | A/environment/Bangladesh/15105/2012(H5N1) | 74.53939 | 31.00304 | 18-Jan-12 |
|  |  |  |  |  |  |  |
|  | 20 | AB684252 | A/chicken/Miyazaki/10/2011(H5N1) | 69.85153 | 38.24020 | Feb-11 |
|  |  | AB747161 | A/peregrine falcon/Miyazaki/22M771/2011(H5N1) | 68.12188 | 37.03830 | 15-Feb-11 |
|  |  | AB747135 | A/peregrine falcon/Miyazaki/22M684/2011(H5N1) | 69.85153 | 37.03830 | 2-Feb-11 |
|  |  |  |  |  |  |  |
|  |  |  |  |  |  |  |
| **H7N9** | 1 | CY147148 | A/environment/Shanghai/S1438/2013(H7N9) | 50.61318 | 44.19611 | 3-Apr-13 |
|  |  | CY147188 | A/pigeon/Shanghai/S1423/2013(H7N9) | 51.11281 | 43.71347 | 3-Apr-13 |
|  |  | CY147132 | A/environment/Shanghai/S1436/2013(H7N9) | 50.61318 | 43.71347 | 3-Apr-13 |
|  |  |  |  |  |  |  |
|  | 2 | KF542876 | A/chicken/Shanghai/017/2013(H7N9) | 52.00389 | 43.08626 | Apr-13 |
|  |  | KF667751 | A/environment/Guangdong/30/2013(H7N9) | 52.27365 | 43.67379 | 26-Apr-13 |
|  |  | KF667746 | A/environment/Guangdong/25/2013(H7N9) | 52.00389 | 43.67379 | 26-Apr-13 |
|  |  |  |  |  |  |  |
|  | 3 | KF667746 | A/environment/Guangdong/25/2013(H7N9) | 52.00389 | 43.67379 | 26-Apr-13 |
|  |  | CY147060 | A/duck/Anhui/SC702/2013(H7N9) | 52.19855 | 44.34205 | 16-Apr-13 |
|  |  | CY147044 | A/chicken/Zhejiang/SD019/2013(H7N9) | 52.00389 | 44.34205 | 11-Apr-13 |
|  |  |  |  |  |  |  |
|  | 4 | KF007116 | A/Nanjing/6/2013(H7N9) | 52.00389 | 44.40570 | 11-Apr-13 |
|  |  | KF007140 | A/Suzhou/5/2013(H7N9) | 51.19473 | 44.59936 | 12-Apr-13 |
|  |  | CY147108 | A/environment/Shandong/SD039/2013(H7N9) | 52.00389 | 44.59936 | 3-May-13 |
|  |  |  |  |  |  |  |
|  | 5 | KF034887 | A/environment/Suzhou/14/2013(H7N9) | 52.27365 | 44.33733 | 4-Apr-13 |
|  |  | CY146908 | A/chicken/Guangdong/SD641/2013(H7N9) | 52.21082 | 44.34205 | 3-May-13 |
|  |  | CY147036 | A/chicken/Zhejiang/SD007/2013(H7N9) | 52.27365 | 44.34205 | 22-Apr-13 |
|  |  |  |  |  |  |  |
|  | 6 | KF007076 | A/Wuxi/4/2013(H7N9) | 53.69294 | 44.33733 | 7-Apr-13 |
|  |  | KF420297 | A/Changsha/2/2013(H7N9) | 53.71343 | 44.34205 | 29-Apr-13 |
|  |  | KF001519 | A/environment/Hangzhou/34/2013(H7N9) | 53.69294 | 44.34205 | 4-Apr-13 |
|  |  |  |  |  |  |  |
|  | 7 | CY147148 | A/environment/Shanghai/S1438/2013(H7N9) | 50.61318 | 44.19611 | 3-Apr-13 |
|  |  | CY147164 | A/homing pigeon/Jiangsu/SD184/2013(H7N9) | 52.31365 | 43.71347 | 20-Apr-13 |
|  |  | CY147132 | A/environment/Shanghai/S1436/2013(H7N9) | 50.61318 | 43.71347 | 3-Apr-13 |
|  |  |  |  |  |  |  |
